# Fostering Undergraduate Medicine, Nursing, and Pharmacy Students’ Readiness for Interprofessional Learning Using High Fidelity Simulation

**DOI:** 10.7759/cureus.12571

**Published:** 2021-01-08

**Authors:** Thomas M Southall, Sandra MacDonald

**Affiliations:** 1 Faculty of Medicine, Memorial University of Newfoundland, St. John's, CAN; 2 School of Nursing, Memorial University of Newfoundland, St. John's, CAN

**Keywords:** interprofessional education, teamwork, high fidelity

## Abstract

Background

Interprofessional education is directly linked to high-quality patient care, however, it remains unclear whether senior undergraduate medicine, nursing, and pharmacy students are ready for interprofessional education using high fidelity human patient simulators.

Purpose

The purpose of this study was to explore student’s readiness for interprofessional learning and determine whether participation in high fidelity interprofessional education resulted in higher levels of readiness for interprofessional learning.

Methods

An interventional program starting with a pre-test before the program and a post-test after the program ends were designed with 24 students. The students were assigned to seven interprofessional teams. Each team participated in a high fidelity interprofessional education module designed to teach the clinical management of an adult patient experiencing acute anaphylaxis. The Readiness for Interprofessional Learning Scale (RIPLS) was used as the pre and post-test instrument.

Results

Prior to participation, students reported a high level of readiness for interprofessional learning, but that readiness significantly improved after participation, including more positive attitudes towards teamwork, enhanced communication skills, and improved respect and trust for team members.

Conclusions

The findings from this study show a higher level of readiness for high fidelity interprofessional learning using human patient simulators among senior undergraduate medicine, nursing, and pharmacy students. These findings support the integration of high fidelity interprofessional education into undergraduate medicine, nursing, and pharmacy undergraduate education programs.

## Introduction

It has been well documented that interprofessional education is an effective teaching and learning strategy often used in undergraduate medicine, nursing, and pharmacy education programs with positive impacts of participation [1-5]. However, few studies have addressed whether high fidelity interprofessional education has any impact on readiness for interprofessional learning. A study by Bolesta and Chmil demonstrated that nursing and pharmacy students’ attitudes towards interprofessional education were measured and the findings indicate that after participation in interprofessional education using human patient simulators, students’ attitudes towards teamwork and collaboration significantly improved [4]. Meanwhile, Mutwali et al. found a positive attitude of nursing, anesthesia, and midwifery students towards teamwork after participation in high fidelity interprofessional education. Students also reported an increased understanding of their roles and responsibilities associated with both their own profession and the profession of other members of the team [6].

These findings were echoed in a study conducted by Scherer et al. that explored the impact of medicine and nursing students’ participation in high fidelity interprofessional education with human patient simulators on attitudes towards teamwork [7]. Findings from that study showed that participation helped to increase student’s understanding of the roles and responsibilities associated with their own and other health professionals’ roles on the interprofessional team. These studies show that participation in high fidelity interprofessional education enhances positive attitudes towards teamwork, but few studies have explored whether participation in high fidelity interprofessional education enhances readiness for interprofessional learning.

Readiness for interprofessional learning

High fidelity simulation experiences provide an exciting learning opportunity for students to work together in a collaborative environment, yet it remains unclear whether students are ready for participation in such activities. Human patient simulators (HPS) are computerized mannequins that replicate real-life patients and are an effective teaching and learning approach to engage learners [8]. HPS appeals to educators because it helps to create a realistic learning environment for students without the risk of live-patient harm. As students work through a case centered around the realism of using HPS, clinical decision making becomes paramount [9]. Students learn to manage a dynamic case where the patient is responding to their actions in real-time. Not only does the HPS respond in real-time, but so do all members of the team. Educators can create realistic simulations with HPS that challenge students from various healthcare backgrounds to work together, just as they would when they graduate. However, integrating HPS into interprofessional education can be costly and requires faculty expertise as well as student orientation to the HPS.

Thom et al. assessed medicine, nursing, and pharmacy students’ readiness for interprofessional learning, with a particular focus on patient safety in the intensive care unit, although it was a case-based discussion and not high fidelity [10]. The study followed a pre-test, post-test design, which required participants to fill out questionnaires prior to and following an interprofessional team discussion of various cases. Results showed that post participation in the case-based discussions, all students had a higher readiness for interprofessional learning and an increased interest in teamwork. Participants also reported increased confidence in their role and increased proficiency while working within the health care team [10]. This study shows that participation in interprofessional education can enhance medicine, nursing, and pharmacy students’ readiness for interprofessional learning, but no insight was gained into whether students are ready for high fidelity interprofessional learning.

Guay et al. examined readiness for high fidelity interprofessional learning in a team of practicing staff members and undergraduate nursing and medical students, using an obstetric-related interprofessional high fidelity education simulation [11]. Students reported a significant positive change in their perception of teamwork, communication, and personal professional identity. Although not all participants were undergraduate students, results fall in-line with the emerging trend that high fidelity interprofessional learning increases participant interest in and perception of teamwork and enhances students’ understanding of their role on the interprofessional healthcare team.

These findings are similar to a study by Murphy and Nimmagadda that reported nursing and social work students scored significantly higher in their readiness for interprofessional learning following a high fidelity clinical simulation [5]. Although limited in number, the findings from these studies suggest that participation in high fidelity interprofessional education can enhance readiness for interprofessional learning but further research is warranted. Therefore, there is a need to examine whether students are ready to participate in high fidelity interprofessional education and whether participation enhances their readiness for interprofessional learning.

Purpose

The purpose of this study was to measure student’s readiness for interprofessional learning and assess the effect of participation in high fidelity interprofessional education on their readiness for interprofessional learning.

Research Questions

1. Are undergraduate medicine, nursing, and pharmacy students ready for interprofessional learning? 2. Does participation in high fidelity interprofessional education result in a higher level of readiness for interprofessional learning?

## Materials and methods

Methodology

**Figure 1:**
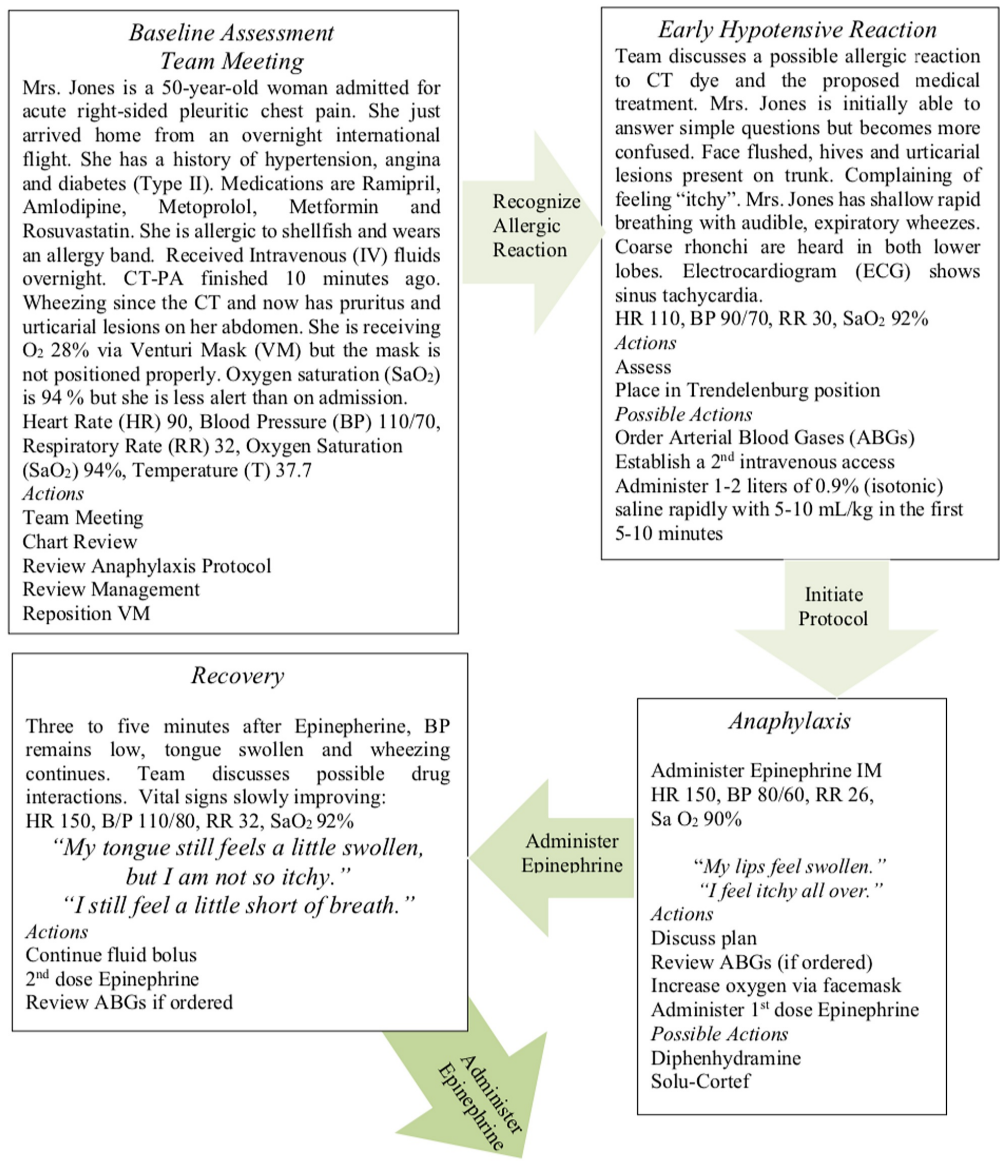
Anaphylaxis Story Board Figure adapted from MacDonald et al. [12].

An interventional program starting with a pre-test before the program and a post-test after the program ends were designed with 24 students. The students were assigned to seven interprofessional teams. Once consent was obtained, participants were given a pre-test Readiness for Interprofessional Learning Scale (RIPLS) before the intervention program, and immediately after participation. The simulation consisted of a 30 min pre-briefing session (including an orientation to the human patient simulator), a one-hour clinical simulation, and a 30-minute debriefing session [12]. The scenario for the simulation was based on managing the care of a patient experiencing acute anaphylaxis. During the simulation, students had access to a “mock” chart containing the patient's admission history and physical, diagnostic reports, nursing notes, nursing care plan, physician’s orders, policies and procedures, medication administration records, and other documents as needed. As the simulation progressed, the patient deteriorated to test the teams response and the team managed care including the administration of a bolus of epinephrine and cardiac monitoring (Figure 1).

Sample

A convenience sampling procedure was done to recruit the participants for the study and consisted of undergraduate nursing (n = 11), medicine (n = 9), and pharmacy (n = 4) students. The students were divided into seven interprofessional teams with a minimum of at least two professions represented on each team. All senior-year students enrolled full time in undergraduate medicine, nursing, or pharmacy program were eligible to participate and over 96 students indicated their interest to participate in the study. However, due to scheduling difficulties, only 24 students could arrange their schedules to participate in the study. One of the barriers to interprofessional education is the complex and fixed nature of health sciences education programs, which complicates scheduling students for interprofessional education.

Setting

The study took place at the Cahill Simulation Room located at the Memorial University of Newfoundland, Faculty of Nursing. The setting resembled a standard patient room in an emergency department in an acute care hospital setting. Pre-briefing and debriefing sessions occurred in a classroom setting.

Instrument

The pre- and post-readiness for interprofessional learning was measured using a valid and reliable instrument, the RIPLS [2,10,13-15]. Parsell and Bligh originally developed the RIPLS to measure the readiness of interprofessional students on four domains including teamwork and collaboration, professional identity or roles of the team, professional responsibilities, and readiness for interprofessional learning [16]. The RIPLS questionnaire includes 19 questions rated on a Likert scale ranging from “five = strongly agree” to “one = strongly disagree”. The RIPLS is a useful and valid tool for collecting data on readiness to learn and is an appropriate instrument for use in this study.

Ethical Considerations

This study received full ethics approval from the Human Research Ethics Board, of Newfoundland, Canada. Participants provided informed consent and understood they could withdraw from the study at any time without prejudice.

Data Analysis

Data from the RIPLS were analyzed as one group (n = 24) using the SPSS data analysis software. The group mean scores of each of the 19 items on the RIPLS were calculated and analyzed using a one-way (ANOVA) to determine whether there were any significant differences in group mean scores from pre-test to post-test. Tukey’s honestly significant differences (HSD) post hoc analysis was performed to confirm significant differences between means.

## Results

Findings

All of the students demonstrated a high level of readiness for interprofessional learning prior to participation in the simulation, as evidenced by the pretest means scores (Table 1). Following participation in the simulation, ten out of the nineteen groups mean scores significantly showed significant improvement in positive attitudes towards teamwork and collaboration, reflecting a better understanding of clinical problems, enhanced communication skills, increased understanding of professional limitations, more trust and respect in the team, and the benefits of interprofessional learning opportunities (Table 1).

**Table 1:** Attitudes, Knowledge, and Readiness for Interprofessional Learning ^a^Scale of “one = strongly disagree to five = strongly agree. ^b^Post hoc analysis Tukey's honestly significant difference (HSD).

Rate your agreement with the following statements^a^	Pre	Post	P-value^b^
Interprofessional learning will increase my ability to understand clinical problems	4.50 ± 0.59	4.83 ± 0.48	.001
Interprofessional learning will help me to understand my own professional limitations	4.50 ± 0.59	4.75 ± 0.44	.013
Learning between healthcare students would improve working relationships	4.67 ± 0.48	4.92 ± 0.66	.038
For small group learning to work students need to respect and trust each other	4.58 ± 0.58	4.88 ± 0.34	.002
I don’t want to waste time learning with other healthcare students	1.57 ± 0.51	1.29 ± 0.46	.024
It is not necessary for undergraduate health care students to learn together	1.57 ±. 0.59	1.39 ± .058	.000
Clinical problem solving can only be learned effectively with students from my own discipline	1.67 ± 0.15	1.63 ± .88	.001
Interprofessional learning will help me to communicate better with patients and other professionals	4.54 ± 0.51	4.92 ± 0.28	.001
I would welcome the opportunity to work on smaller group projects with other healthcare students	4.21 ± 0.78	4.46 ± 0.66	.000
I would welcome the opportunity to share some generic lectures, tutorials, or workshops with other healthcare students	4.21 ± 0.78	4.54 ± 0.66	.000
Interprofessional learning and practice will help me to clarify the nature of patient’s or clients’ problems	4.25± 0.74	4.58 ± 0.65	.000
Interprofessional learning will help me to become a better team worker	4.50± 0.52	4.75 ± 0.44	.003
I have to acquire much more knowledge and skill than other students in my own discipline	2.54 ± 1.02	2.38 ± 1.09	.003

Teamwork and Collaboration

Teamwork and collaboration involve valuing interprofessional learning and respecting students from other healthcare professions. These findings indicate that students’ attitudes towards teamwork and collaboration significantly improved after participation in the simulation. After participation, students reported an increased ability to understand clinical problems, which is especially important when interprofessional teams are managing patients with rapidly changing conditions such as acute anaphylaxis. One student commented “Great experience! I think it is really helpful for understanding communication and roles.” When managing complex clinical problems, students need to recognize the importance of communicating and collaborating as a team. After participating in the high fidelity interprofessional simulation, students also reported significantly improved respect for and trust of the other members of the team. Working with the team to understand complex clinical problems, while maintaining respect and trust are important characteristics of effective health care teams.

Professional Identify and Roles of the Team

When members of the interprofessional team are aware of their own professional identity and understand the roles of the other team members, teamwork and collaboration are enhanced. The findings from this study indicate that after participation in the simulation, students reported a significant improvement in their understanding of their own professional role and the role of the other members of the team. This is in keeping with the findings from the literature that reported high fidelity interprofessional education helped to increase student’s understanding of the roles and responsibilities associated with their own and other health care professionals on the team. 

Students also reported they were more supportive of interprofessional learning as a means of improving the working relationships of the team. This would indicate students recognized the importance of interprofessional learning as a way to improve teamwork and collaboration. Students also reported a significant improvement in their understanding that interprofessional learning will help them to become better team member and improve working relationships on the team. These findings indicate that participation in the simulation not only enhanced students’ understanding of their own role on the team but also helped them to understand the role of the other members of the team.

Professional Responsibilities

Students reported significantly more positive attitudes toward the practical application of professional skills with other healthcare professional students, after participation in the simulation. Attitudes towards team communication improved, indicating the experience had helped them to better understand the importance of team communication especially when caring for a deteriorating patient. Another area of improved attitudes was in the students’ own understanding of their professional limitations, e.g., scope of practice. This would indicate that participation in the high fidelity interprofessional simulation enhanced students’ understanding of their professional responsibilities including their own professional limitations within their scope of practice.

Readiness for Learning

Readiness for interprofessional learning refers to an openness to participate in learning experiences with other health care students and an understanding of the beneficial outcomes of interprofessional learning. After participation in the simulation, students reported a significant improvement in positive attitudes towards working with other health care students on small group projects, as well as attitudes towards sharing generic lectures, tutorials, or workshops. One student commented, “Wish we had more opportunities for interprofessional learning! Maybe simulated rounds?” and another student stated, “We need way more!” These findings indicate that students are ready for high fidelity interprofessional learning and would welcome the opportunity to share learning experiences with other health care students.

## Discussion

The purpose of this study was to answer two questions: are undergraduate medicine, nursing, and pharmacy students ready for high fidelity interprofessional education, and does participation in high fidelity interprofessional education result in a higher level of readiness for interprofessional learning. The findings from this study suggest that all of the participants were ready for interprofessional learning and would welcome the opportunity to share more opportunities for interprofessional learning through lectures, tutorials, or workshops with other healthcare students. All students agreed that interprofessional learning promoted positive attitudes towards interprofessional teamwork and helped them to become better members of the team. This is in keeping with current research that supports interprofessional education is an effective teaching and learning strategy to foster teamwork and collaboration in undergraduate health sciences students.

This study adds to the growing body of research that supports the use of high fidelity interprofessional education in undergraduate medicine, nursing, and pharmacy programs to foster teamwork and collaboration. However, the small sample in this study limits the generalizability of the findings, therefore more research is needed to confirm health sciences students are ready for interprofessional learning using human patient simulators and to provide guidance on how to integrate that learning into health sciences undergraduate education. The challenges associated with scheduling students to participate in this study also confirms that one of the major barriers to interprofessional education at the undergraduate level is the difficult task of coordinating student schedules to learn together. The complex and fixed nature of medicine, nursing, and pharmacy students’ schedules is a critical limitation for integrating high fidelity interprofessional education into undergraduate education, but the findings from this study indicate it could be worth the effort.

## Conclusions

Senior undergraduate medicine, nursing, and pharmacy students were ready for interprofessional learning and demonstrated improved positive attitudes towards teamwork and collaboration after participation in high fidelity interprofessional education using human patient simulators. Further research is warranted to confirm these findings and provide guidance for the integration of high fidelity interprofessional education into all levels of undergraduate medicine, nursing, and pharmacy education programs.
